# Supplementary material for: Comparative efficacy and acceptability of interventions for insomnia in breast cancer patients: A protocol for systematic review and network meta-analysis
Source: PLoS One. 2023 Mar 7;18(3):e0282614. doi: 10.1371/journal.pone.0282614 (PMC9990914; doi:10.1371/journal.pone.0282614)
Supplement: S1 Appendix — (DOCX) [file pone.0282614.s001.docx]

## **Appendix 1: Search strategy for each database**

**PubMed**

#1 "Randomized Controlled Trial"[Publication Type] OR "Controlled Clinical Trial"[Publication Type] OR "Clinical Trials as Topic"[Mesh] OR randomized[Title/Abstract] OR randomised[Title/Abstract] OR randomly[Title/Abstract] OR placebo[Title/Abstract] OR trial[Title]

#2 "Animals"[Mesh] NOT "Humans"[Mesh]

#3 #1 NOT #2

#4 "Sleep Initiation and Maintenance Disorders"[Mesh] OR "Sleep Wake Disorders"[Mesh] OR "Wakefulness"[Mesh] OR "Sleepiness"[Mesh] OR "Sleep Stages"[Mesh] OR dyssomni*[Title/Abstract] OR insomn*[Title/Abstract] OR sleep*[Title/Abstract] OR Wakeful*[Title/Abstract]

#5 "Breast Neoplasms"[Mesh] OR "Breast Carcinoma In Situ"[Mesh] OR "Breast Neoplasms, Male"[Mesh] OR "Carcinoma, Ductal, Breast"[Mesh] OR "Carcinoma, Lobular"[Mesh] OR "Inflammatory Breast Neoplasms"[Mesh] OR "Triple Negative Breast Neoplasms"[Mesh] OR "Unilateral Breast Neoplasms"[Mesh] OR breast neoplasm*[Title/Abstract] OR breast tumor*[Title/Abstract] OR breast carcinoma*[Title/Abstract] OR breast cancer*[Title/Abstract] OR breast tumour*[Title/Abstract] OR mammary neoplasm*[Title/Abstract] OR mammary tumor*[Title/Abstract] OR mammary carcinoma*[Title/Abstract] OR mammary cancer*[Title/Abstract] OR mammary tumour*[Title/Abstract] OR breast adenocarcinoma*[Title/Abstract] OR breast carcinogenesis[Title/Abstract] OR breast sarcoma*[Title/Abstract] OR phyllodes tumor*[Title/Abstract] OR intraductal carcinoma*[Title/Abstract] OR lobular carcinoma*[Title/Abstract]

#6 #3 AND #4 AND #5

**EMBASE**

#1 'randomized controlled trial'/exp OR 'controlled clinical trial'/exp OR 'randomization'/exp OR 'randomized':ab,ti OR 'randomised':ab,ti OR 'randomly':ab,ti OR 'clinical trial (topic)'/exp OR 'placebo'/exp OR 'placebo':ab,ti OR 'trial':ab,ti

#2 'animal'/exp NOT 'human'/exp

#3 #1 NOT #2

#4 'insomnia'/exp OR 'sleep disorder'/exp OR 'wakefulness'/exp OR 'sleep stage'/exp OR 'dyssomni*':ab,ti OR 'insomn*':ab,ti OR 'sleep*':ab,ti OR 'Wakeful*':ab,ti

#5 'breast tumor'/exp OR 'breast carcinoma in situ'/exp OR 'Paget nipple disease'/exp OR 'lobular carcinoma'/exp OR 'inflammatory breast cancer'/exp OR 'triple negative breast cancer'/exp OR 'breast neoplasm*':ab,ti OR 'breast tumor*':ab,ti OR 'breast carcinoma*':ab,ti OR 'breast cancer*':ab,ti OR 'breast tumour*':ab,ti OR 'mammary neoplasm*':ab,ti OR 'mammary tumor*':ab,ti OR 'mammary carcinoma*':ab,ti OR 'mammary cancer*':ab,ti OR 'mammary tumour*':ab,ti OR 'breast adenocarcinoma*':ab,ti OR 'breast carcinogenesis':ab,ti OR 'breast sarcoma*':ab,ti OR 'phyllodes tumor*':ab,ti OR 'intraductal carcinoma*':ab,ti OR 'lobular carcinoma*':ab,ti

#6 #3 AND #4 AND #5

**Cochrane Library**

#1 MeSH descriptor: [Sleep Initiation and Maintenance Disorders] explode all trees

#2 MeSH descriptor: [Sleep Wake Disorders] explode all trees

#3 MeSH descriptor: [Wakefulness] explode all trees

#4 MeSH descriptor: [Sleepiness] explode all trees

#5 MeSH descriptor: [Sleep Stages] explode all trees

#6 (dyssomni*):ti,ab,kw OR (insomn*):ti,ab,kw OR (sleep*):ti,ab,kw OR (Wakeful*):ti,ab,kw

#7 #1 OR #2 OR #3 OR #4 OR #5 OR #6

#8 MeSH descriptor: [Breast Carcinoma In Situ] explode all trees

#9 MeSH descriptor: [Carcinoma, Ductal, Breast] explode all trees

#10 MeSH descriptor: [Carcinoma, Lobular] explode all trees

#11 MeSH descriptor: [Inflammatory Breast Neoplasms] explode all trees

#12 MeSH descriptor: [Triple Negative Breast Neoplasms] explode all trees

#13 MeSH descriptor: [Unilateral Breast Neoplasms] explode all trees

#14 (breast neoplasm*):ti,ab,kw OR (breast tumor*):ti,ab,kw OR (breast carcinoma*):ti,ab,kw OR (breast cancer*):ti,ab,kw OR (breast tumour*):ti,ab,kw OR (mammary neoplasm*):ti,ab,kw OR (mammary tumor*):ti,ab,kw OR (mammary carcinoma*):ti,ab,kw OR (mammary cancer*):ti,ab,kw OR (mammary tumour*):ti,ab,kw OR (breast adenocarcinoma*):ti,ab,kw OR (breast carcinogenesi):ti,ab,kw OR (breast sarcoma*):ti,ab,kw OR (phyllodes tumor*):ti,ab,kw OR (intraductal carcinoma*):ti,ab,kw OR (lobular carcinoma*):ti,ab,kw

#15 #8 OR #9 OR #10 OR #11 OR #12 OR #13 OR #14

#16 #7 AND #15

**PsycINFO**

1 exp Randomized Controlled Trials/

2 exp Clinical Trials/

3 ("Randomized Controlled Trial" OR "Controlled Clinical Trial" OR "Clinical Trials" OR random* OR placebo OR trial).ti. OR ("Randomized Controlled Trial" OR "Controlled Clinical Trial" OR "Clinical Trials" OR random* OR placebo OR trial).ab.

4 1 OR 2 OR 3

5 limit 4 to human

6 exp Sleep Wake Disorders/

7 exp Wakefulness/

8 exp Sleepiness/

9 exp Insomnia/

10 ("Sleep Initiation and Maintenance Disorders" OR "Sleep Wake Disorders" OR "Wakefulness" OR "Sleepiness" OR "Sleep Stages" OR dyssomni* OR insomn* OR sleep* OR Wakeful*).ti. OR ("Sleep Initiation and Maintenance Disorders" OR "Sleep Wake Disorders" OR "Wakefulness" OR "Sleepiness" OR "Sleep Stages" OR dyssomni* OR insomn* OR sleep* OR Wakeful*).ab.

11 6 OR 7 OR 8 OR 9 OR 10

12 exp Breast Neoplasms/

13 (breast neoplasm* or breast tumor* or breast carcinoma* or breast cancer* or breast tumour* or mammary neoplasm* or mammary tumor* or mammary carcinoma* or mammary cancer* or mammary tumour* or breast adenocarcinoma* or "breast carcinogenesis" or breast sarcoma* or phyllodes tumor* or intraductal carcinoma* or lobular carcinoma*).mp.

14 12 OR 13

15 5 AND 11 AND 14

**Web of Science**

#1 TS=("Randomized Controlled Trial" OR "Controlled Clinical Trial" OR "Clinical Trials" OR random*)

#2 TS="Animals" NOT TS="Humans"

#3 #1 NOT #2

#4 TS=("Sleep Initiation and Maintenance Disorders" OR "Sleep Wake Disorders" OR "Wakefulness" OR "Sleepiness" OR "Sleep Stages" OR dyssomni* OR insomn* OR sleep* OR Wakeful*)

#5 TS=(breast neoplasm* OR breast tumor* OR breast carcinoma* OR breast cancer* OR breast tumour* OR mammary neoplasm* OR mammary tumor* OR mammary carcinoma* OR mammary cancer* OR mammary tumour* OR breast adenocarcinoma* OR "breast carcinogenesis" OR breast sarcoma* OR phyllodes tumor* OR intraductal carcinoma* OR lobular carcinoma*)

#6 #3 AND #4 AND #5
