# Supplementary material for: Comparative efficacy and acceptability of interventions for insomnia in breast cancer patients: A protocol for systematic review and network meta-analysis
Source: PLoS One. 2023 Mar 7;18(3):e0282614. doi: 10.1371/journal.pone.0282614 (PMC9990914; doi:10.1371/journal.pone.0282614)
Supplement: Attachment — Submitted filename: Response to Reviewers.docx [file pone.0282614.s003.docx]

PONE-D-22-24531

Comparative efficacy and acceptability of interventions for insomnia in breast cancer patients: A protocol for systematic review and network meta-analysis

PLOS ONE

Dear Dr. Andrea Giannini,

We have received and reviewed the editors' and reviewers’ comments on our manuscript: *Comparative efficacy and acceptability of interventions for insomnia in breast cancer patients: A protocol for systematic review and network meta-analysis.* Thank you very much for the opportunity to revise our manuscript. A point-by-point response to the comments follows. We highlighted all the changes made to the original manuscript.

My colleagues and I are grateful for your consideration of our work, and we look forward to hearing your decision.

Junqiang Lei

Dear Dr. Jun-Qiang Lei

Thank you for submitting your manuscript to PLOS ONE. After careful consideration, we feel that it has merit but does not fully meet PLOS ONE’s publication criteria as it currently stands. Therefore, we invite you to submit a revised version of the manuscript that addresses the points raised during the review process.

Dear authors,
the topic of the present article titled “Comparative efficacy and acceptability of interventions for insomnia in breast cancer patients: A protocol for systematic review and network meta-analysis” is very interesting, the paper and the aim falls within the scope of the journal but the article needs major improvements.
Review some spellings. Methods and results section and discussion should be modified and improved.
I suggest improving the manuscript with the reviewers' comments

Please submit your revised manuscript by two weeks. If you will need more time than this to complete your revisions, please reply to this message or contact the journal office at [plosone@plos.org](mailto:plosone@plos.org). When you're ready to submit your revision, log on to https://www.editorialmanager.com/pone/ and select the 'Submissions Needing Revision' folder to locate your manuscript file.

Please include the following items when submitting your revised manuscript:

- A rebuttal letter that responds to each point raised by the academic editor and reviewer(s). You should upload this letter as a separate file labeled 'Response to Reviewers'.
- A marked-up copy of your manuscript that highlights changes made to the original version. You should upload this as a separate file labeled 'Revised Manuscript with Track Changes'.
- An unmarked version of your revised paper without tracked changes. You should upload this as a separate file labeled 'Manuscript'.

If you would like to make changes to your financial disclosure, please include your updated statement in your cover letter. Guidelines for resubmitting your figure files are available below the reviewer comments at the end of this letter.

If applicable, we recommend that you deposit your laboratory protocols in protocols.io to enhance the reproducibility of your results. Protocols.io assigns your protocol its own identifier (DOI) so that it can be cited independently in the future. For instructions see:<https://journals.plos.org/plosone/s/submission-guidelines#loc-laboratory-protocols>. Additionally, PLOS ONE offers an option for publishing peer-reviewed Lab Protocol articles, which describe protocols hosted on protocols.io. Read more information on sharing protocols at <https://plos.org/protocols?utm_medium=editorial-email&utm_source=authorletters&utm_campaign=protocols>.

**Reply:** Thank you very much for all these instructions. We revised our manuscript carefully.

Journal Requirements:

When submitting your revision, we need you to address these additional requirements.

1. Please ensure that your manuscript meets PLOS ONE's style requirements, including those for file naming. The PLOS ONE style templates can be found at 
https://journals.plos.org/plosone/s/file?id=wjVg/PLOSOne_formatting_sample_main_body.pdf and 
https://journals.plos.org/plosone/s/file?id=ba62/PLOSOne_formatting_sample_title_authors_affiliations.pdf

2. We note that you have stated that you will provide repository information for your data at acceptance. Should your manuscript be accepted for publication, we will hold it until you provide the relevant accession numbers or DOIs necessary to access your data. If you wish to make changes to your Data Availability statement, please describe these changes in your cover letter and we will update your Data Availability statement to reflect the information you provide.

3. PLOS requires an ORCID iD for the corresponding author in Editorial Manager on papers submitted after December 6th, 2016. Please ensure that you have an ORCID iD and that it is validated in Editorial Manager. To do this, go to ‘Update my Information’ (in the upper left-hand corner of the main menu), and click on the Fetch/Validate link next to the ORCID field. This will take you to the ORCID site and allow you to create a new iD or authenticate a pre-existing iD in Editorial Manager. Please see the following video for instructions on linking an ORCID iD to your Editorial Manager account: https://www.youtube.com/watch?v=_xcclfuvtxQ

**Reply:** Thank you very much for all these instructions. We revised our manuscript carefully.

Reviewers' comments:

Reviewer's Responses to Questions

**Comments to the Author**

1. Does the manuscript provide a valid rationale for the proposed study, with clearly identified and justified research questions?

The research question outlined is expected to address a valid academic problem or topic and contribute to the base of knowledge in the field.

Reviewer #1: Yes

Reviewer #2: Yes

Reviewer #3: No

Reviewer #4: Partly

2. Is the protocol technically sound and planned in a manner that will lead to a meaningful outcome and allow testing the stated hypotheses?

The manuscript should describe the methods in sufficient detail to prevent undisclosed flexibility in the experimental procedure or analysis pipeline, including sufficient outcome-neutral conditions (e.g. necessary controls, absence of floor or ceiling effects) to test the proposed hypotheses and a statistical power analysis where applicable. As there may be aspects of the methodology and analysis which can only be refined once the work is undertaken, authors should outline potential assumptions and explicitly describe what aspects of the proposed analyses, if any, are exploratory.

Reviewer #1: Yes

Reviewer #2: Yes

Reviewer #3: Yes

Reviewer #4: Partly

3. Is the methodology feasible and described in sufficient detail to allow the work to be replicable?

Descriptions of methods and materials in the protocol should be reported in sufficient detail for another researcher to reproduce all experiments and analyses. The protocol should describe the appropriate controls, sample size calculations, and replication needed to ensure that the data are robust and reproducible.

Reviewer #1: Yes

Reviewer #2: Yes

Reviewer #3: Yes

Reviewer #4: Yes

4. Have the authors described where all data underlying the findings will be made available when the study is complete?

The [PLOS Data policy](https://journals.plos.org/plosone/s/materials-and-software-sharing) requires authors to make all data underlying the findings described in their manuscript fully available without restriction, with rare exception, at the time of publication. The data should be provided as part of the manuscript or its supporting information, or deposited to a public repository. For example, in addition to summary statistics, the data points behind means, medians and variance measures should be available. If there are restrictions on publicly sharing data—e.g. participant privacy or use of data from a third party—those must be specified.

Reviewer #1: Yes

Reviewer #2: No

Reviewer #3: Yes

Reviewer #4: Yes

5. Is the manuscript presented in an intelligible fashion and written in standard English?

PLOS ONE does not copyedit accepted manuscripts, so the language in submitted articles must be clear, correct, and unambiguous. Any typographical or grammatical errors should be corrected at revision, so please note any specific errors here.

Reviewer #1: Yes

Reviewer #2: Yes

Reviewer #3: Yes

Reviewer #4: Yes

**6. Review Comments to the Author**

Please use the space provided to explain your answers to the questions above and, if applicable, provide comments about issues authors must address before this protocol can be accepted for publication. You may also include additional comments for the author, including concerns about research or publication ethics.

You may also provide optional suggestions and comments to authors that they might find helpful in planning their study.

(Please upload your review as an attachment if it exceeds 20,000 characters)

**Reviewer #1:** I think the article is very interesting and current. From our knowledge we know that insomnia is determined not only by the diagnosis of the tumor but also by its treatment. it happens very often that after an almost complete recovery, hormone therapy exacerbates a previous insomnia or creates alterations in the sleep wake cycle de novo. I believe it is essential to stress this speech in the introduction and discussion.

**Reply:** Thank you very much for your positive comments and good suggestion. We have revised the “Introduction” and “Discussion” sections accordingly.

**Introduction Section**

“For breast cancer patients, in addition to the stress caused by the cancer diagnosis, some treatment-related factors are often related to insomnia. Vasomotor symptoms, including hot sweating and night sweats, are common with chemotherapy-induced amenorrhea and endocrine therapies and can cause insomnia episodes or aggravate pre-existing conditions [11-14].”

**Discussion Section**

“Approximately 30%-50% of breast cancer survivors have insomnia persisting years after treatment [52]. Stress, anxiety, discomfort after surgery such as chronic pain after breast surgery, and side effects of treatment such as hot sweats and night sweats, which are common after chemotherapy and endocrine therapy, can all contribute to insomnia in breast cancer patients [12, 14, 52, 54, 55].”

**Reviewer #2**: Question 4 - The authors have stated that all relevant data from the study will be made available upon study completion; this is a protocol, not a completed study.

**Reply:** Thank you for pointing this out. We have revised this statement.

- The study will explore the "comparative efficacy and acceptability" of interventions for insomnia in breast cancer patients. Efficacy of interventions will be able to be shown once the data is analyzed; however, what is meant by "acceptability" and how will that be explored and analyzed?

**Reply:** Thank you for pointing this out. We have refined this in the manuscript.

“To measure the acceptability of treatments, we will use the proportion of patients who stopped treatment for any reason (all cause discontinuation) [42].”

“We will use pooled odds ratio (OR) with 95% credible intervals for the dichotomous variables (ie. acceptability).”

- It is surprising that there will be "...no restriction on the language of publication" of studies to be selected. Will there be translation help furnished, or are the authors knowledgable in a large number of languages?

**Reply:** Thank you for pointing this out. We have added the sentence “For publications that are not in English and Chinese, we will seek translation assistance” to our manuscript.

- The controls will be "...waiting list, treatment/care as usual, etc." Do the authors mean to assemble control patients, find controls outside of the selected articles, or use the "controls" who are presented in the RCTs used?

**Reply:** Thank you for pointing this out. We will investigate the potential clinical and statistical heterogeneity of non-interventional controls to determine whether to assemble control patients.

“The controls will include waiting list, treatment/care as usual, placebos. We will investigate the potential clinical and statistical heterogeneity of non-interventional controls (waiting list, treatment/care as usual, placebo) to determine whether to treat them as a single node.”

- It is surprising that, out of seven authors, only two will screen all articles and review all the selected articles. Are the remaining authors to be writers and perform analytics only?

**Reply:** Thank you for pointing this out. We revised “two reviewers” to “pairs of reviewers".

**Reviewer #3:** Unfortunately, I do not think it is interesting for us to know the ongoing protocol of review studies.

**Reply:** On behave of the authors, I wish to express my profound gratitude to you for finding time out of your busy schedule to review this manuscript. Our research will explore the comparative effectiveness and acceptability of all currently available interventions for insomnia in patients with breast cancer, and we will use the GRADE approach to assess the certainty of the evidence supporting the effect of treatment. The state-of-the-art methodology will also be used to summarize the relative effectiveness of competing interventions. The results of our review will help provide more evidence for the treatment of insomnia in breast cancer patients.

**Reviewer #4:** The elegibility criteria should be explained more accurately and there is no stratification of patients

**Reply:** Thank you for the suggestion. We refined the eligibility criteria and added subgroup analyses based on patient stratification.

**“Eligibility criteria**

**Types of studies**

Randomized controlled trials comparing the effects of different interventions on the management of insomnia in breast cancer patients will be included. RCTs should report at least one measure outcome of sleep and provide enough detail to calculate effect sizes. We will exclude cluster or crossover RCTs.

**Population**

Individuals with a breast cancer diagnosis who had clinically relevant levels of insomnia as defined by a standardized diagnostic system such as the Diagnostic and Statistical Manual of Mental Disorders (DSM) [39], International Classification of Sleep Disorders (ICSD) [40], Tenth revision of the International Statistical Classification of Diseases and Health-Related Problems (ICD-10) [41], and other well-recognized classifications. We will put no restrictions on the subtype, histology, stage, and treatment of breast cancer, age and comorbidities of patients, and duration of insomnia.

**Intervention**

Any pharmacological and non-pharmacological interventions used for the management of insomnia in breast cancer patients. The eligible interventions are listed in Table 1.

**Comparator**

The controls will include waiting list, treatment/care as usual, placebo, or a different pharmacological or non-pharmacological intervention. We will investigate the potential clinical and statistical heterogeneity of non-interventional controls (waiting list, treatment/care as usual, placebo) to determine whether to treat them as a single node.

**Outcome**

Outcomes of interest are sleep efficiency (SE, %), total sleep time (TST), sleep onset latency (SOL), wake after sleep onset (WASO), acceptability. SE, SOL, WASO, and TST are typically defined as a ratio of time asleep to time in bed, the length of time after lights out until sleep onset, the length of waking time after the onset of persistent sleep, and total nighttime sleep, respectively. In addition, the scores from the Insomnia Severity Index (ISI) or the Pittsburgh Sleep Quality Index (PSQI) are also the outcomes that we are interested in. For both questionnaires, a higher score indicates more severe insomnia (ISI) or worse sleep quality (PSQI). To measure the acceptability of treatments, we will use the proportion of patients who stopped treatment for any reason (all cause discontinuation) [42].”

**“Subgroup analysis and sensitivity analysis**

Where sufficient data are available, we will conduct subgroup analyses or meta-regression analyses based on country and mean age of patients; subtype, histology, stage, and treatment (eg, surgery, chemotherapy, endocrine therapy) of breast cancer; comorbid disorders, and duration of interventions and insomnia. We will also conduct sensitivity analyses to explore the influence of variables on the outcomes. Planned sensitivity analyses will exclude trials with a high risk of bias or significant levels of missing data.”

7. PLOS authors have the option to publish the peer review history of their article ([what does this mean?](https://journals.plos.org/plosone/s/editorial-and-peer-review-process#loc-peer-review-history)). If published, this will include your full peer review and any attached files.

If you choose “no”, your identity will remain anonymous but your review may still be made public.

**Do you want your identity to be public for this peer review?** For information about this choice, including consent withdrawal, please see our [Privacy Policy](https://www.plos.org/privacy-policy).

Reviewer #1: **Yes:** Dott. Alessandro De Luca

Reviewer #2: No

Reviewer #3: No

Reviewer #4: No
