# Supplementary material for: Comparative efficacy and acceptability of interventions for insomnia in breast cancer patients: A protocol for systematic review and network meta-analysis
Source: PLoS One. 2023 Mar 7;18(3):e0282614. doi: 10.1371/journal.pone.0282614 (PMC9990914; doi:10.1371/journal.pone.0282614)
Supplement: Attachment — Submitted filename: Response to Reviewers.docx [file pone.0282614.s004.docx]

PONE-D-22-24531R1

Comparative efficacy and acceptability of interventions for insomnia in breast cancer patients: A protocol for systematic review and network meta-analysis

PLOS ONE

Dear Dr. Andrea Giannini,

We have received and reviewed the editors' and reviewers’ comments on our manuscript: *Comparative efficacy and acceptability of interventions for insomnia in breast cancer patients: A protocol for systematic review and network meta-analysis.* Thank you very much for the opportunity to revise our manuscript. A point-by-point response to the comments follows. We highlighted all the changes made to the original manuscript.

My colleagues and I are grateful for your consideration of our work, and we look forward to hearing your decision.

Junqiang Lei

Dear Dr. Junqiang,

Thank you for submitting your manuscript to PLOS ONE. After careful consideration, we feel that it has merit but does not fully meet PLOS ONE’s publication criteria as it currently stands. Therefore, we invite you to submit a revised version of the manuscript that addresses the points raised during the review process.

Please submit your revised manuscript by Feb 20 2023 11:59PM. If you will need more time than this to complete your revisions, please reply to this message or contact the journal office at [plosone@plos.org](mailto:plosone@plos.org). When you're ready to submit your revision, log on to https://www.editorialmanager.com/pone/ and select the 'Submissions Needing Revision' folder to locate your manuscript file.

Please include the following items when submitting your revised manuscript:

- A rebuttal letter that responds to each point raised by the academic editor and reviewer(s). You should upload this letter as a separate file labeled 'Response to Reviewers'.
- A marked-up copy of your manuscript that highlights changes made to the original version. You should upload this as a separate file labeled 'Revised Manuscript with Track Changes'.
- An unmarked version of your revised paper without tracked changes. You should upload this as a separate file labeled 'Manuscript'.

If you would like to make changes to your financial disclosure, please include your updated statement in your cover letter. Guidelines for resubmitting your figure files are available below the reviewer comments at the end of this letter.

If applicable, we recommend that you deposit your laboratory protocols in protocols.io to enhance the reproducibility of your results. Protocols.io assigns your protocol its own identifier (DOI) so that it can be cited independently in the future. For instructions see: <https://journals.plos.org/plosone/s/submission-guidelines#loc-laboratory-protocols>. Additionally, PLOS ONE offers an option for publishing peer-reviewed Lab Protocol articles, which describe protocols hosted on protocols.io. Read more information on sharing protocols at <https://plos.org/protocols?utm_medium=editorial-email&utm_source=authorletters&utm_campaign=protocols>.

**Reply:** Thank you very much for all these instructions. We revised our manuscript carefully.

Journal Requirements:

Please review your reference list to ensure that it is complete and correct. If you have cited papers that have been retracted, please include the rationale for doing so in the manuscript text, or remove these references and replace them with relevant current references. Any changes to the reference list should be mentioned in the rebuttal letter that accompanies your revised manuscript. If you need to cite a retracted article, indicate the article’s retracted status in the References list and also include a citation and full reference for the retraction notice.

Additional Editor Comments:

Dear authors,
the manuscript it has now been evaluated by our experts and they have recommended that minor changes be made to the submission.
Please improving the manuscript with the reviewers' comments.

**Reply:** Thank you very much for all these instructions. We revised our manuscript carefully.

Reviewers' comments:

Reviewer's Responses to Questions

**Comments to the Author**

1. Does the manuscript provide a valid rationale for the proposed study, with clearly identified and justified research questions?

The research question outlined is expected to address a valid academic problem or topic and contribute to the base of knowledge in the field.

Reviewer #1: Yes

Reviewer #2: Yes

Reviewer #3: Yes

2. Is the protocol technically sound and planned in a manner that will lead to a meaningful outcome and allow testing the stated hypotheses?

The manuscript should describe the methods in sufficient detail to prevent undisclosed flexibility in the experimental procedure or analysis pipeline, including sufficient outcome-neutral conditions (e.g. necessary controls, absence of floor or ceiling effects) to test the proposed hypotheses and a statistical power analysis where applicable. As there may be aspects of the methodology and analysis which can only be refined once the work is undertaken, authors should outline potential assumptions and explicitly describe what aspects of the proposed analyses, if any, are exploratory.

Reviewer #1: Yes

Reviewer #2: Yes

Reviewer #3: Partly

3. Is the methodology feasible and described in sufficient detail to allow the work to be replicable?


Descriptions of methods and materials in the protocol should be reported in sufficient detail for another researcher to reproduce all experiments and analyses. The protocol should describe the appropriate controls, sample size calculations, and replication needed to ensure that the data are robust and reproducible.

Reviewer #1: Yes

Reviewer #2: Yes

Reviewer #3: Yes

4. Have the authors described where all data underlying the findings will be made available when the study is complete?

The [PLOS Data policy](https://journals.plos.org/plosone/s/materials-and-software-sharing) requires authors to make all data underlying the findings described in their manuscript fully available without restriction, with rare exception, at the time of publication. The data should be provided as part of the manuscript or its supporting information, or deposited to a public repository. For example, in addition to summary statistics, the data points behind means, medians and variance measures should be available. If there are restrictions on publicly sharing data—e.g. participant privacy or use of data from a third party—those must be specified.

Reviewer #1: Yes

Reviewer #2: Yes

Reviewer #3: Yes

5. Is the manuscript presented in an intelligible fashion and written in standard English?

PLOS ONE does not copyedit accepted manuscripts, so the language in submitted articles must be clear, correct, and unambiguous. Any typographical or grammatical errors should be corrected at revision, so please note any specific errors here.

Reviewer #1: Yes

Reviewer #2: Yes

Reviewer #3: Yes

6. Review Comments to the Author

Please use the space provided to explain your answers to the questions above and, if applicable, provide comments about issues authors must address before this protocol can be accepted for publication. You may also include additional comments for the author, including concerns about research or publication ethics.

You may also provide optional suggestions and comments to authors that they might find helpful in planning their study.


(Please upload your review as an attachment if it exceeds 20,000 characters)

**Reviewer #1:** I think the article is very interesting and current. It is very important for clinical practice to investigate issues such as insomnia in women diagnosed with breast cancer. This study certainly addresses an issue that needs more study and therefore more attention.

**Reply:** Thank you very much for your positive comments.

**Reviewer #2:** - Although the authors did answer all the questions from the reviewers and stated how they changed the text, they did not provide a version of the manuscript with tracked changes. Thus, it was extremely difficult to find where the text had been changed; reviewers could only see what the rewrite was by itself.

**Reply:** Thank you very much for your comments. We have provided two versions of manuscript: a version of manuscript with marked changes; an unmarked version of our revised paper without tracked changes.

- In several places, the term "hot sweating" was used as one of the vasomotor symptoms. I believe that this is used instead of "hot flashes" or "hot flushes"? This should be changed.

**Reply:** Thank you for pointing this out. We have changed "hot sweating" to "hot flashes" across our manuscript.

- It is still not very clear what "acceptability" means; I believe it is meant how well the intervention was accepted by patients and whether they continued to use the intervention because it was effective. Could this be clarified?

**Reply:** Thank you for your good suggestion. We revised our manuscript accordingly.

“To measure the acceptability of treatments, we will use the proportion of patients who stopped treatment for any reason (all-cause discontinuation). Acceptability reflects how well the intervention was accepted by patients and whether they continued to use the intervention because it was effective.”

**Reviewer #3:** The results of this study will attract physician's interest. I hope this study will provide some evidence for the management of the insomnia in breast cancer patients.

**Reply:** Thank you very much for your interest in our study.

7. PLOS authors have the option to publish the peer review history of their article ([what does this mean?](https://journals.plos.org/plosone/s/editorial-and-peer-review-process#loc-peer-review-history)). If published, this will include your full peer review and any attached files.


If you choose “no”, your identity will remain anonymous but your review may still be made public.


**Do you want your identity to be public for this peer review?** For information about this choice, including consent withdrawal, please see our [Privacy Policy](https://www.plos.org/privacy-policy).

Reviewer #1: No

Reviewer #2: No

Reviewer #3: No
